# Design and Implementation of an Interactive Web-Based Near Real-Time Forest Monitoring System

**DOI:** 10.1371/journal.pone.0150935

**Published:** 2016-03-31

**Authors:** Arun Kumar Pratihast, Ben DeVries, Valerio Avitabile, Sytze de Bruin, Martin Herold, Aldo Bergsma

**Affiliations:** Laboratory of Geo-Information Science and Remote Sensing, Wageningen University, P.O. Box 47, 6700 AA, Wageningen, The Netherlands; DOE Pacific Northwest National Laboratory, UNITED STATES

## Abstract

This paper describes an interactive web-based near real-time (NRT) forest monitoring system using four levels of geographic information services: 1) the acquisition of continuous data streams from satellite and community-based monitoring using mobile devices, 2) NRT forest disturbance detection based on satellite time-series, 3) presentation of forest disturbance data through a web-based application and social media and 4) interaction of the satellite based disturbance alerts with the end-user communities to enhance the collection of ground data. The system is developed using open source technologies and has been implemented together with local experts in the UNESCO Kafa Biosphere Reserve, Ethiopia. The results show that the system is able to provide easy access to information on forest change and considerably improves the collection and storage of ground observation by local experts. Social media leads to higher levels of user interaction and noticeably improves communication among stakeholders. Finally, an evaluation of the system confirms the usability of the system in Ethiopia. The implemented system can provide a foundation for an operational forest monitoring system at the national level for REDD+ MRV applications.

## 1. Introduction

Tropical forests play an important role in stabilizing the climate, providing food, water, wood products and provide habitats for biodiversity [1]. Deforestation and forest degradation are now widely acknowledged by the scientific community as major contributors to recent increases in atmospheric greenhouse gas (GHG) concentrations and changes to the world's hydrological cycle [2, 3]. To reduce atmospheric GHG concentrations, the United Nations Framework Convention on Climate Change (UNFCCC) has proposed an international carbon trade mechanism, Reducing Emissions from Deforestation and Degradation (REDD+), to support the reduction of emissions and to enable forest conservation, sustainable management of forests and the enhancement of forest carbon stocks in developing countries [4]. Aside from being an important step towards reducing GHG concentrations, REDD+ also includes considerations for co-benefits, safeguards and biodiversity protection [5–7].

Several bilateral and multilateral efforts, such as the World Bank administered Forest Carbon Partnership Facility (FCPF), the UN Collaborative Programme on Reducing Emissions from Deforestation and Forest Degradation in Developing Countries (UN-REDD Programme), and the Norwegian International Climate and Forests Initiative are currently supporting developing countries to prepare Readiness Preparation Proposals for the implementation of REDD+ projects at the national level [8–10]. One of the main tasks for countries participating in REDD+, as requested by the UNFCCC (Decision 2/CP.19) [11], is to develop an operational, robust, transparent and cost-effective national forest monitoring system (NFMS) that supports measuring, reporting and verification (MRV) of actions and achievements of REDD+ activities [12–14].

Currently most forest monitoring focuses on activity data, i.e. data on forest cover changes [13], and two approaches are used: top-down and bottom-up. The top-down approach utilizes satellite systems [15, 16] whereas the bottom-up approach utilizes ground observation through government agencies [17], community-based monitoring (CBM) [18], participatory monitoring [19] or volunteered geographic information [20]. Satellite data provide systematic coverage and a higher frequency of acquisition at a low cost, which is crucial for near real-time (NRT) forest monitoring [16, 21]. Recently, efforts have been made to establish an operational time-series based NRT forest monitoring system [22]. These efforts include the use of optical remote sensing satellites such as MODIS [23, 24] and Landsat [25]. NRT systems contribute to better forest management, allowing governments and local stakeholders to take action to avoid or reduce illegal activities and enhancing transparency in the use of forest resources. However, the operational use of these systems are influenced by several factors such as cloud cover, seasonality and the limited spatial, spectral and temporal resolution of satellite observations that lead to inevitable lag in forest change detection [16, 26]. Furthermore, existing systems are not capable of providing information about forest degradation and regrowth, and do not consider community involvement in ground verification, validation and law-enforcement activities.

Bottom-up ground observations have traditionally been produced, analysed, and disseminated by trained experts, often from government agencies. The major drawbacks of bottom-up data are that they are expensive, often not NRT and therefore are not fit for REDD+ MRV needs [14, 27]. In the last few years, CBM has become popular in REDD+ countries as a way to increase local participations and engagements in forest monitoring and management processes [27–30]. Several transparent, logical, feasible and repeatable methods have been proposed by researchers to demonstrate that communities can contribute to 1) forest carbon stock measurements and emission factor assessments [28, 31–33] and 2) forest change monitoring (activity data quantification) [31, 34, 35]. Because of communities’ presence on the ground, they are able to signal forest change activities (deforestation, forest degradation or reforestation) and provide information such as location, time, size and proximate drivers of the change events on an NRT basis [35–37]. Modern electronic communication devices, such as smartphones, have simplified efforts in data collection and transmission [31, 38, 39]. However, issues have arisen when integrating CBM data into NFMS including: 1) lack of confidence in the data collection procedure, 2) inconsistent monitoring frequency, 3) limited spatial coverage, 4) variable data quality and 5) lack of trust of data providers [35, 40–42]. Recent advances in technologies like Web 2.0, GIS, remote sensing, big data processing, mobile devices and social media, on the other hand, have provided possible solutions to these issues [39, 43, 44].

In the past, two independent approaches have been used to monitor the forest of UNESCO Kafa Biosphere Reserve, Ethiopia: remote sensing analysis [45] and community-based monitoring [35]. Both approaches have shown advantages and disadvantages but neither of the approaches were comprehensive enough to monitor all types of forest change (ie. deforestation, forest degradation and reforestation). In the first approach, dense Landsat Normalized Difference Vegetation Index (NDVI) time-series were used to monitor small-scale deforestation with high accuracy [45]. However, it had limitations in monitoring forest degradation. In the second approach, CBM using local experts was found to be more reliable in monitoring forest degradation with spatial, temporal and thematic details [35]. Nevertheless, these data had limited spatial coverage, and a lack of consistency in monitoring frequency and temporal accuracy in deforestation detection [35]. Hence, effective monitoring will likely require an integrated approach, where detailed community-based observations are combined with remote sensing satellites [35]. Compared to traditional systems, an interactive forest monitoring system (IFMS) offers some immediate advantages:

1. Up-to-date information on forest change location, size, timing and drivers of forest change which are consistent over time.
2. Cost-effective and sustainable data collection due to open source software and open data policies.
3. Transparent results which can be integrated into to NFMS/national MRV systems.
4. Potential to enhance participation of stakeholders in forest monitoring and management.

Despite this potential, the effective development of interactive NRT forest monitoring system is currently lacking due to several reasons. Firstly, there are no operational methods to analyse forest change information from multisource data streams (i.e. Satellite and CBM in NRT). Secondly, there is no system that can systematically store, visualize and provide access to the forest change information via the internet to local stakeholders. Thirdly, there is a lack of spatial and temporal forest change search query capabilities. Lastly, interaction between users and the system is generally “passive”, in the sense that individuals do not receive feedback on the submitted data. These limitations have motivated us to develop an IFMS which uses Web-GIS as its integrating platform. We have used knowledge from distinct areas of research on public participation Geographic Information Systems (PPGIS) [46], environmental monitoring through Web-GIS [47], community-based monitoring [27], agricultural fields planning [48], satellite based NRT forest and fire monitoring [49–52], spatial data infrastructure [53], social networking [54, 55] and open source technologies [56]. The aforementioned research and technologies demonstrate that developing a satellite-community forest monitoring systems is possible, as technologies to support such system are mature. In addition, web mapping platforms together with mobile technologies [57] offer a unique opportunity to integrate satellite data and community-based observations to monitor forest disturbances, whereby the participation of the local stakeholders in forest monitoring is ensured. However, implementation challenges need to be explored and evaluated on a case-by-case basis. In particular, open data policies [58], big data processing environments [44], and advancement in satellite time-series methods [45, 59] should be considered.

The objectives of this study are 1) to design an interactive system that combines Web-GIS technologies, satellite and CBM data, and social media to support near real-time forest monitoring, 2) to implement and operationalize the developed system in the UNESCO Kafa Biosphere Reserve in Southwestern Ethiopia and 3) to assess the usability of the system in Kafa.

## 2. Material and Methods

### 2.1 Study Area and Project Context

The study site is located in the Kafa Zone (7.22°E to 7.84°E and 35.59°N to 37.17°N), Southern Nations Nationalities and People’s Region (SNNPR), in Southwestern Ethiopia, covering an area of 700,000 ha. The Kafa region was recognized as a Biosphere Reserve within UNESCO’s Man and the Biosphere program in March 2011. It has a seasonal climate with annual rainfall of around 1700 mm and a rainy season lasting from June to September. The altitude ranges from 400 to 3100 m with an average annual temperature of 19°C. A large portion (~50%) is still covered by Afromontane cloud forests [60]. The region is also recognised as an important gene bank of Coffee Arabica [61] and of many other endemic species of plants, mammals and birds. Although the forest has been protected through UNESCO’s conservation programs, still there are many challenges that have threatened its ecological coherence. Subsistence agriculture, human settlements expansion, industrial coffee plantations, and domestic firewood and charcoal extractions are recognised as the major drivers of deforestation that has negative impact on the integrity of the reserve [35, 62].

The proposed study was conducted within the framework of a project implemented by the Nature and Biodiversity Conservation Union (NABU). The Kafa Zone Bureau of Agriculture and Rural Development, Participatory Forest Management (PFM) groups, and Woreda and Kebele (local administrative levels) authorities were main Stakeholders of this project. Under a collaborative scheme of monitoring [19], one local expert from each of the ten Woredas was recruited. These local experts had at least a basic education background and some fundamental understanding of forest monitoring. A mobile device (Samsung GT-S7710) equipped with a global positioning system (GPS) receiver was provided to each of these local experts to acquire forest monitoring data. These experts also had other responsibilities such as biodiversity monitoring, development of ecotourism infrastructure, reforestation, community plantations and awareness-raising for the sustainable use of forest resources (e.g., honey and wild coffee).

### 2.2 Ethical Statement

Prior to beginning of the study, approval was obtained from both Laboratory of Geo-information Science and Remote Sensing—Wageningen University, The Netherlands and NABU scientific advisors. During the research design and implementations, ethical standards in the treatment of participants have been maintained and implemented in accordance with binding UNESCO Man and the Biosphere (MAB) regulations, Ethiopian Government guideline of participatory forest management (PFM) programs, BirdLife regulations (Convention No. 169) as well as the Ethiopian constitution (e.g. Policy for women and indigenous peoples). Personal information was not collected from participants during this study and participants were able to withdraw from the study at any time. At the end, At the end, written permission were obtained from all participant and NABU for the publications.

### 2.3 System Architecture

Our web-based IFMS was designed in a collaborative manner with local stakeholders to assist them in identifying forest change locations in NRT and to facilitate the optimal allocation of information about the changes [35, 47, 50, 63]. The functional features of the system include: 1) functionality to download, store and process NRT forest change detection using Landsat time-series images, 2) facility to upload ground observations and download forest change locations on demand, 3) ability to map and show the hotspots of forest changes in space-time, 4) capability to provide feedback to local stakeholders and 5) functionality to provide interaction through social media.

**Fig 1:**
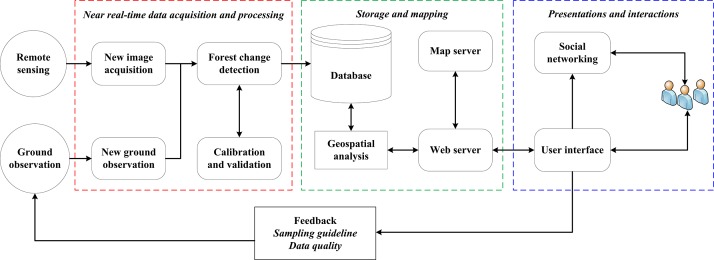
Diagram of the interactive web-based near real-time forest monitoring system.

Multi-layered software architectures are commonly used for the development of distributed, dynamic, flexible and re-configurable we-based service systems that can meet the service requirements of many different users [64]. In this research, we implemented multi-layered software architecture in a modular fashion. Fig 1 summarizes the general framework that was adopted in the design of the IFMS. The design of the IFMS was organized into four functional modules: 1) NRT data acquisition and processing, 2) storage and mapping, 3) presentation and interaction and 4) feedback. The above-mentioned four modules comprise the primary server side functionality. On the client side, users (local stakeholders and forest professionals in charge of monitoring and decision making) send requests to the server via a Mobile device or Web-based graphic user interface (web-browser). The server carries out the corresponding spatial analysis and conveys the results to the client for feedback, interaction and visualization. The framework is scalable and extensible so that additional functions or map layers of external data sources can be easily added to the application.

### 2.4 System implementation

Each of the modules was developed using open source tools (Table 1) and source codes are available through the following link https://github.com/pratihast/Interactive-forest-monitoring-system. We described the individual modules in detail below:

1. Data entry was facilitated by a decision-based data acquisition form using open data kit (ODK) [31, 35]. The form rendered on an Android platform through ODK Collect, which allows multiple data records to be entered (text variables, GPS position, photo etc.) and stored on a mobile device. After data collection, users transferred the collected data to the database server through a general packet radio service (GPRS) message via 2G, 3G or other networks.
2. A semi-automated process chain was developed to download, store and process Landsat image time-series. All the historic as well as continuously acquired Landsat TM, ETM+ and OLI surface reflectance images with WRS-2 coordinates path 170 and row 55 at processing level LT1 were obtained from the United States Geological Survey (USGS) Earth Resources Observation and Science (EROS) Center Science Processing Architecture (ESPA, http://espa.cr.usgs.gov). After downloading the Landsat data, multiple pre-processing steps were applied: application of the FMASK algorithm [65] for cloud masking, computation of NDVI and application of a forest mask. We used the BFAST Monitor method [49] for breakpoint detection in Landsat NDVI time-series images for NRT forest change detection.
3. The spatial database structure was designed to allow for different types of data, including basic geographic data, ground observation data and remote sensing information, to be stored, managed and accessed through structured query language (SQL). Basic geographic data included the geometry of Woredas, Kebeles and boundaries of the Biosphere Reserve. We used PostgreSQL with PostGIS extensions to implement our design. Stored datasets were published using Geo-server as a Web Map Service (WMS), which is compliant with Open Geospatial Consortium (OGC) specifications. We used OpenLayers and JQuery JavaScript libraries to provide client side functionality to display and render maps in web pages.
4. A graphical user interface was developed using Hyper Text Markup Language (HTML), Cascading Style Sheets (CSS) and Hypertext Preprocessor (PHP). In addition to this, we also provided dynamic spatiotemporal query and social media plugins features. These features allow users to generate forest change locations on-demand, to download forest change locations in usable GPS exchange format (GPX) format, which can directly be used on a GPS device, and to efficiently communicate results using social media sites. The “Near Real-Time Disturbance Monitoring—Kafa Biosphere Reserve” Facebook group was created to provide a platform for posts, discussions and comments regarding the results.

**Table 1:** Open source tools used for the development of the interactive web-based near real-time forest monitoring system.

Open source tools	Version	Function	Source
ODK Design		Decision based ground data acquisition form design	https://opendatakit.org/help/form-design/
ODK Collect	1.4.5	Renders forms into a sequence	http://opendatakit.org
ODK Aggregate	1.3.2	Deploy data into server	http://opendatakit.org
Bulk Download Application	1.1.4	Downloading Landsat imagery	http://earthexplorer.usgs.gov/bulk/
R	2.14.1	Time-series analysis	http://r-project.org
BFASTSpatial		Time-series analysis	http://dutri001.github.io/bfastSpatial/
PostgreSQL	9.1	Database	http://postgresql.org
PostGIS	2.0.6	Spatial extension for PostgreSQL	http://postgis.org
Apache	2.2.22	Web server	http://httpd.apache.org
GeoServer	6.0.3	Web mapping server	http://geoserver.org
OpenLayers	1.12	Frontend web mapping library	http://openlayers.org
jQuery	1.8	Frontend JavaScript library	http://jquery.org
PhP	5.4.36	Web development	http://php.net/

### 2.5 System Evaluation

Several approaches have been proposed by researchers to evaluate Web-GIS systems [50, 66, 67]. Most of these approaches are focused on technological aspects rather than usability aspects [68]. In this study, we emphasize the usability aspects of Web-GIS system in terms of “fitness for purpose”. Fitness for purpose concerns the degree to which a system fits the users’ needs, thus bringing the utility of the system closer to the users requirements [69]. The evaluation framework of the IFMS is shown in Table 2 and comprised a three-step process: 1) identifying the purpose, 2) exploring the indicators associated with the purpose and 3) finding out an appropriate data source for each indicator. Each of these steps were performed in a systematic way to perceive ease of use of the offered system.

**Table 2:** Indicators used for the evaluation of interactive web-based near real-time forest monitoring system in context of REDD+.

***Purpose 1*: *stakeholder participation and interaction in forest monitoring process***		
**Evaluation criterion**	**Indicators**	**Source**
Training and capacity-building activities	Number of training and capacity events	Training and capacity events after the launch of the system *[Source*: Institutional record*]
Training and capacity-building activities	Number of participants	Registration forms of participant *[Source*: *Institutional record* *]
Use of services	Number of visitors of the system	System view statistics Multiple requests from the same IP address are counted as one view *[Source*: *System*^†^ *log analysis]*
Engagement in debate and knowledge sharing	Number of users in social media page	User statistics Facebook group *[Source*: *Facebook group*^‡^*]*
Engagement in debate and knowledge sharing	Number of post/feed in the social media page	Post statistics Facebook group *[Source*: *Facebook group*^‡^*]*
Engagement in debate and knowledge sharing	Responses to posts	Post seen, like and comments Percentage of user engagements = [Number of user seen+ Number of user like+ Number of user comments on the post] / Total number of user*100 *[Source*: *Facebook group*^‡^*]*
*Purpose 2*: *Provide up-to-date and accurate information on forest change which are consistent over time*, *and can be integrated into the national forest monitoring system/ national MRV system*		
Ground-based forest monitoring alerts	Number of ground observations alerts	Near real-time ground-based forest monitoring *[Source*: *ground observation datasets]*
Satellite based forest change alerts	Number of satellite based forest change alerts	Near real-time satellite based forest change alerts concerning patches larger than 0.5 ha *[Source*: *Landsat time-series analysis]*
Consistency of ground-based observations and satellite based alerts	Number of spatio-temporal coincidence	Number of ground observation associated to identified satellite based alerts (within the radius of 1 km) *[Source*: *ground observation datasets]*
Consistency of ground-based observations and satellite based alerts	Percentage of thematic agreement	Percentage of agreements (‘true’, ‘success’) or disagreement (‘false’, ‘failure’) of a series of satellite based alerts visited by local experts *[Source*: *ground observation datasets and Landsat time-series alerts]*
*Purpose 3*: *Increase awareness and law enforcement*		
Law enforcement	Number of illegal activities	Illegal activities reported by local experts *[Source*: *ground observation datasets]*
Awareness	Awareness approach	List of Awareness program *[Source*: Institutional record*]

*Institution:- NABU project office, Kafa Ethiopia (http://www.kafa-biosphere.com/)

*†*System:-Web-based interactive near real-time forest monitoring system (www.cbm.wur.nl)

^‡^Facebook group:-Near Real-Time Disturbance Monitoring—Kafa Biosphere Reserve (https://www.facebook.com/groups/kafa.forest.monitoring/)

In the first step, the purpose of the IFMS design was reviewed. The main purposes of IFMS was 1) to ensure stakeholder participation and interaction in forest monitoring process, 2) to provide up-to-date and accurate information on forest change which are consistent over time that can be integrated into NFMS/national MRV system and 3) to increase awareness and law enforcement. These purpose were identified primarily with forest monitoring objectives in mind, but were also compliant with REDD+ MRV requirements [19, 27, 35, 70]. In the second step, the set of evaluation criteria of the IFMS was defined. After defining the evaluation criteria of the system, a list of 12 indicators associated with each criteria was compiled. The evaluation criteria served as a necessary intermediary link between purpose and indicators, enabling a more systematic and coherent evaluation of the system. In the third step, we employed both qualitative and quantitative research approaches to achieve the relevant information. First, we established email communication with NABU project co-ordinator (the qualitative approach) to gather all relevant and existing records on IFMS systems and their uses as perceived by different stakeholders in Kafa Biosphere. Second, the quantitative source of information was obtained from ground-based forest monitoring alerts provided by local experts and forest change alerts obtained from Landsat analysis. Furthermore, user interaction statistics were obtained from social media page and system log analysis.

## 3. Results

### 3.1 Overview of the System

**Fig 2:**
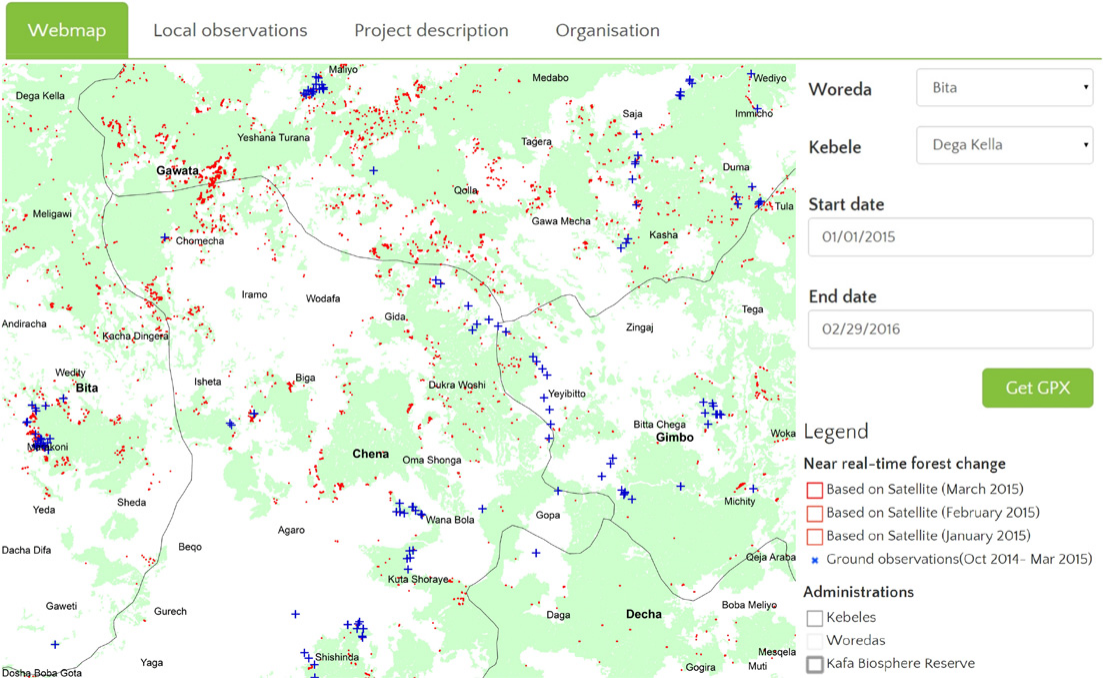
Web interface showing an example of a visualization interface for the Kafa case study. In this example, Forest map derived from Landsat image 2010 is used as base map, forest change polygons derived from Landsat data are displayed in red and local observation points in blue.

The developed web-based IFMS can be accessed at: http://www.cbm.wur.nl. A screenshot of the deployed IFMS is shown in Fig 2. The right panel of Fig 2 shows the mapping interface of the system. This interface provides the overview of the mapping layers and adequate mapping functionalities (e.g., layer selection, zooming and panning facilities). The data layers are organised into three information categories: administrative boundaries, historical forest change and near real-time forest change. By default, the interface shows forest map as background layer overlaid with NRT satellite based forest change alerts (in Red) and ground observation (in Blue), which are also shown in Fig 2. The left panel shows the interface for spatial and temporal querying. A temporal query is defined by a start date and an end date, while a spatial query identifies a location window. The SQL meeting both conditions are selected from the database and are available for download in GPX format. This GPX format helps the local experts to transfer the monitoring alerts on their GPS device. This alerts empower local expert to reach at suspected locations of recent forest change.

**Fig 3:**
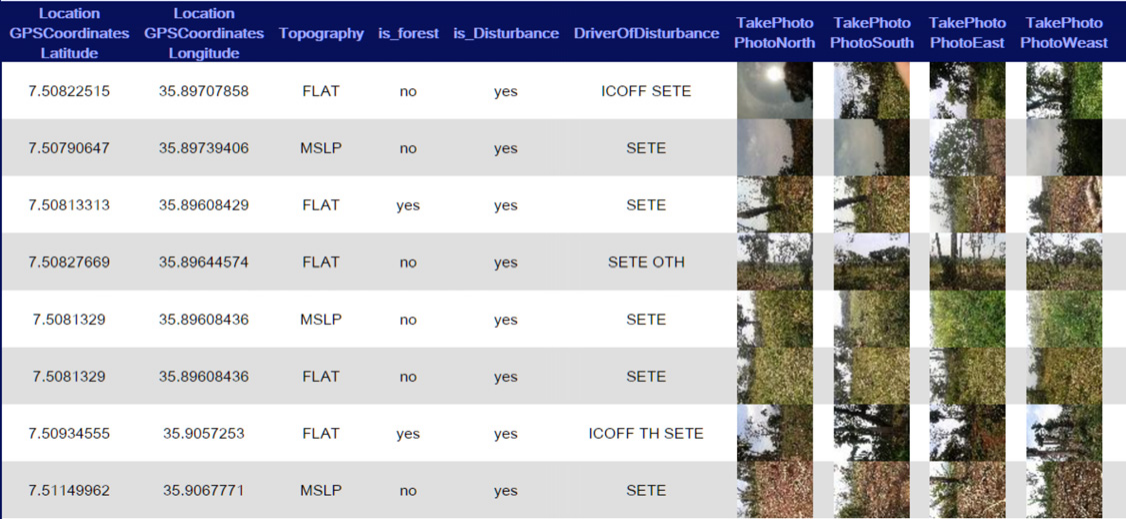
Web interface showing an example of a visualization interface for ground observation collected by local experts. In this example, driver of forest disturbances are Intensive Coffee Cultivation (ICOFF), Settlement Expansion (SETE), Timber Harvesting (TH) and Others (OTH).

Fig 3 exemplifies ground observation data collected by local experts. The screenshot demonstrates that these data contain three categories of information: spatial, temporal and thematic. The spatial category includes GPS location, administrative boundary information of forest change location and the estimated size of forest change. The temporal category includes time of forest change. Finally, thematic category provides the information about Land use type, forest change type, drivers of forest change, photo and multimedia description of the forest change location. These datasets contain some sensitive information such as driver of forest change, photo and multimedia description about the process of change. Hence, access restrictions have been made for the general public, but authorised users can have full access and download facilities to all the attributes of these ground data.

### 3.2 System Evaluation Result

The IFMS was launched in October 2014. The evaluation was conducted for seven months to allow adequate time for the target user community to use the application. We divided these seven months of implementation into three phases: a kick-off phase, a demonstration phase and an operational phase. During the kick-off phase (October 2014), the system was launched and a series of intensive training and capacity building programs were conducted to encourage user participation. In the demonstration phase (November- December 2014), the system was used with some improvements and limited assistance was provided to the users. During the operational phase (January to April 2015), users were able to use the system independently. The following subsections summarize the evaluation result of IFMS under three categories of purpose indicated in Table 2.

The overall evaluation results concerning stakeholder participation and interaction are shown in Table 3. During the kick-off phase of the system, stakeholders had indicated the need for training and capacity-building programs. Hence, a one-week training workshop (27th October 2014 to 3rd November 2014) was conducted in Kafa, Ethiopia. In the demonstration and operational phases, on the other hand, users were already well trained with the implemented system and thus less training and capacity-building efforts were needed. In total, 37 participants participated during the kick-off phase, but the number of participants dropped to 15 during the demonstration and operational phase of the system. The main reason behind this drop is that participants from different organisation such as Kafa Zone Bureau of Agriculture and Rural Development, PFM groups, Woredas, and Kebeles were invited to join the training during the kick-off phase of the system, while only some were directly involved in ground observations.

**Table 3:** System performance for stakeholder participation and interaction in forest monitoring processes.

Indicators	Indicator value (per month)		
	Kick-off phase	Demonstration phase	Operation phase
Number of trainings and capacity-building events	7 days	2 days	1 day
Number of participants in training and capacity-building activities	37	15	15
Number of visitors of the services	4	4	8
Number of users in social media page	24	26	28
Number of post feed on social media page	5	10	15
Percentage of engagement in debate and knowledge sharing	72%	78%	87%

The web log history showing the number of visitors to the IFMS (Table 3) showed that the use of the system in Kafa biosphere reserves doubled in the operational phase compared to the kick-off and demonstration phases. In line with this, the Facebook group user statistics showed a small increment of users during demonstration and operational phase of the system. The number of Facebook posts increased by factor of 2 in the demonstration phase and by factor of 3 in the operational phase of the system (Table 3). The average users' engagements in each of the posts also increased throughout this implementation phase of the system.

The evaluation results concerning up-to-date and accurate information on forest change are summarized in Table 4. Local experts uploaded many NRT ground observation alerts during the kick-off phase of the system. The numbers of ground observation decreased in the demonstration phase while they slightly increased during the operational phase of the system. These fluctuations could be due to topography, seasonality and weather conditions. In contrast, the number of satellite based NRT forest change alerts (representing areas greater than 0.5 ha) increased to 88 locations per month during the operational phase of the system. The number of spatio-temporal coincidence analysis shows that more than 50% of the local monitoring reports were recorded within 1 km radius of identified hotspot sites. The thematic agreement results (Table 4) show that the accuracy of satellite based forest change alerts improved to 77% during the operational phase.

**Table 4:** System performance for near real-time information on forest change.

Indicator	Indicator value (per month)		
	Kick-off phase	Demonstration phase	Operation phase
Number of ground observation alerts	173	103	114
Satellite based near real-time forest change alerts	27	20	88
Number of spatio-temporal coincidence	90	65	62
Percentage of thematic agreement	74%	71%	77%

Increased awareness was evaluated based on two aspects. The first aspect involved a decrease in the number of illegal activities. During the Kick-off phase of the system, 22 incidents were reported. This number decreased by almost 50% in the subsequent phases, where 15 events were reported in the demonstration phase and 12 in the operational phase. The second aspect involved two modes of public awareness events that were organized to increase awareness of locals about forest protection. On 17^th^ March 2015 there was a 30-minute radio program on the Kafa Community Radio to disseminate information about the forest disturbance alerts and protection efforts. Secondly, awareness meetings were conducted by spiritual leaders in communities throughout the Biosphere reserve. In total 447 peoples participated in these meetings. Details on the awareness programs are provided in S1 Appendix.

## 4. Discussion

### 4.1 Reflection on Design and Implementation of the System

Several authors [23, 71, 72] and some governmental initiatives (e.g., Brazil Detecção de Desmatamento em Tempo Real (DETER) [73]) have emphasized the importance of combining technologies, services and data sources to build credible and legitimate NRT forest monitoring system at the appropriate scale, either at local, provincial or national scales. These systems provide information about deforestation hotspots. Such information has made it possible for law enforcement, civil society, and the media to react to illegal activities quickly and reduce the rate of deforestation [74, 75]. However, the reality is that most tropical countries have limited technical and financial capacities to design and operate such systems [14]. In this regard, we have been able to demonstrate the design and implementation of a web-based NRT IFMS and evaluate its usability in the Kafa Biosphere Reserve, Ethiopia.

Compared to existing forest monitoring system, our IFMS differs in the following aspects:

1. Open source-based system: Recently, some efforts have been made towards developing interactive NRT forest monitoring system using open source technologies (e.g. DETER [73], Global Forest Watch http://www.globalforestwatch.org/) but these systems need to be improved by incorporating local data streams to make them more interactive and to ensure the participation of local stakeholders in monitoring their forests. In this regard, our system is able to demonstrate the novel aspects that integrate multiple data sources (satellite and CBM data) and open source technologies in modular fashion (Fig 1, Table 1). Each of these model are functionality independent, such that each modules contains everything necessary to execute one aspect of the desired functionality [64].
2. Spatial coverage: The NRT system such as DETER has been used since 2004 as a part of government plan to control the Amazon deforestation [73]. This system is based on MODIS data and is able to monitor larger scale forest change (Greater than 25 ha) in near real-time (every 15 days). However, this system has spatial limitations which could hinder its implementation in a sub-Saharan context, and especially in Ethiopia, where the forest change occurs on a small scale and is mostly driven by small holder subsistence agriculture [76]. In this regard, our proposed system utilises higher spatial resolution sensor (i.e. 30m in the case of Landsat) and thus the system is able to detect the forest change cluster size greater than 0.6 ha [45].
3. Community participation: Our system engages local communites in NRT ground based forest monitoring. The ability to signal forest degradation and describe the process of change with high temporal detail highlights the advantage of the local approach [35] used in this study over conventional satellite-based NRT system [70,71]. Similar to the previous study conducted by Pratihast et al. [31], the results of this study also show that mobile devices improve local experts’ data acquisition capacities with regards to the location, time, size and type of forest change events.
4. Enhanced stakeholder’s interaction: Our system offers better interaction between the local stakeholders. These stakeholders can contribute data to the server and access the forest change information to support decision making. Rather than just using a monologic transmission model (publishing the forest change alerts on the web), our system utilises dialogic transmission system with interactive means of communication, such as social media. Combining social media and a web interface has increased the information exchange and helped to mitigate problems of interactivity. Hence, this system enables stakeholders to access forest change information, collaborate on common efforts and build relationships amongst each other. These findings confirm the results of many other studies showing the capabilities of social media in effective communication [50, 54, 77].

### 4.2 Critical Review of the System Evaluation Results

In this study, we assessed the usability of the implemented IFMS in terms of fitness for purpose. A number of findings have emerged from this research regarding community-based forest monitoring systems, many of which are new, while others resonate with the findings of previous studies. This section discusses the three aspects of this research in light with the three purposes outlined in subsection 2.4.

Overall the results (Table 3) indicate that the number of participants decreased during the demonstration and operational phase of the system. This issue have also been noticed in previous research of community-based forest monitoring, citizen science and PPGIS research [19, 46]. The number of participants might be improved through the implementation of appropriate collaborative and benefit schemes [19, 78]. Such collaborative schemes could encourage the involvement of other interested organisations and individuals such as local communities, PFM groups, non-governmental organizations and government authorities in IFMS. The benefit schemes, including financial, political (e.g. empowerment, participation in decision making) or indirect benefits, can also increase the participation of local stakeholders [79].

The second aspect is concerned with the integration of satellite and community-based data for forest change monitoring. As exemplified by Claudio et al. [80], our results (Table 4) indicate that the integrated approach helped to improve the spatial, temporal and thematic details of forest change information. We used Landsat satellite data-based alerts as a top-down approach to detect forest change. Similar to the work of DeVries et al. [45], our results (Table 4) show that the Landsat based NDVI time-series method is able to detect small scale forest change alerts in NRT. However, the thematic quality of these alerts vis a vis forest change processes and drivers of change is very limited. Furthermore, the persistent cloud cover over the area induced data gaps and hampered forest change detection. We expect that Landsat 8 and the future Sentinel 2 missions [81] will play a major role in overcoming these limitations. On the other hand, we have utilised CBM, as bottom-up approach to monitor and verify the satellite-based forest change alerts. The results of this study show that the satellite-based alerts were able to mobilize the local experts to visit the targeted locations (Table 4). The ground observation alerts provided by the local experts had high thematic details [35]. However, lack of coverage of the whole study area and consistency of the ground observations present limitations to this approach. These limitations can be addressed by engaging more local experts, volunteers, PFM groups and citizens in monitoring and providing appropriate training and benefit schemes [78].

The final aspect concerns the interaction of stakeholders with the system. The results show that the local stakeholders were more actively engaged in social media than with the IFMS (Table 3). The ability of social media platforms to run in environments of limited internet bandwidth is likely the reason behind the difference in interaction levels between the IFMS and the social media component [77]. This issue might be solved in the future with the improvement of internet coverage. Alternatively, IFMS should take into account low bandwidth situations and should be designed to operate more efficiently in such environments.

### 4.3 Limitations of the System

This paper describes a proof-of-concept study regarding the design and implementation of an IFMS. An initial fitness for purpose evaluation of our implementation has been carried out and the results are being prepared for this publication. Overall, the evaluation results are favourable but more research over the longer time period with more participants is required to realise its true benefits and potentialities in terms of NRT forest monitoring.

At the moment, our IFMS faces two key limitations in terms of design, which introduce some time lag in NRT forest change monitoring. First, the system utilises a semi-automatic process chain for Landsat data processing. Our system utilises a manual approach to search and download newly available Landsat image from USGS server. This manual approach prevents us from establish an automatic process chain for Landsat data. Recently, USGS have improved their data distribution infrastructures and allows automatic download facilities for Landsat scenes through external platforms such as the Google Earth Engine [82]. Second, the IFMS provides manual download functionalities for NRT forest change alerts, implying that users must download the alerts manually from our web-interface. This manual interaction introduces some time lag in disseminating the alerts to the local stakeholders. This limitation could be overcome by sending the forest change alerts directly to mobile devices and utilising geo-fence services to notify people about the forest change locations [83].

## 5. Conclusions

Near real-time forest monitoring is necessary to support good forest management. It allows local stakeholders to take prompt action, which may avoid or reduce illegal activities and enhance transparency in the use of forest resources.

In this research, we describe the design and implementation of an interactive web-based NRT forest monitoring system and its evaluation in the UNESCO Kafa Biosphere Reserve in Southwestern Ethiopia. The proposed IFMS integrates three components: Web-GIS technologies, satellite and CBM data source and social media. The functional features of our IFMS are as follows: 1) functionality to download, store, process and run NRT forest change detection on Landsat time-series images, 2) facility to upload ground observations and download forest change location from the analysis of remote sensing on-demand, 3) ability to map and show hotspots of forest changes in space-time, 4) capability to provide feedback to local stakeholders and 5) functionality to provide interaction through social media. The evaluation results show that the IFMS empowers local experts participation and interaction in forest monitoring processes and provides up-to-date and accurate information on forest change. This information is consistent over time, can be integrated into NFMS/national MRV system, and ultimately enhances the effective communication among the stakeholders for forest management.

## 6. Outlook

Remote sensing satellites, mobile technologies, web-mapping platforms and internet service technologies are constantly improving. These improvements will provide new opportunities for NRT forest monitoring system. A number of future outlooks have been identified with regards to IFMS:

1. Testing over a larger area with more diversified user community is required.
2. Integrating multi-sensor remote sensing data streams such as Landsat, Sentinel 1 and Sentinel 2 mission may improve the forest change detection [72, 81].
3. Developing mobile data acquisition tools and utilising a geo-fence service can enhance the local stakeholders participation in forest monitoring [83].
4. Extension of the system is recommended in the domains of biodiversity and safeguards for National REDD+ implementation.

## Supporting Information

S1 Appendix Forest protection awareness meeting in Kafa, Ethiopia. (DOCX)
