# Supplementary material for: Design and Implementation of an Interactive Web-Based Near Real-Time Forest Monitoring System
Source: PLoS One. 2016 Mar 31;11(3):e0150935. doi: 10.1371/journal.pone.0150935 (PMC4816390; doi:10.1371/journal.pone.0150935)
Supplement: S1 Appendix — (DOCX) [file pone.0150935.s001.docx]

*S1 Appendix. Forest protection awareness meeting in Kafa, Ethiopia*

| **Date** | Kebele | Participants | | |
| --- | --- | --- | --- | --- |
|  |  | Male | Female | Total |
| 15-16 November 2014 | Yeyebito | 61 | 35 | 96 |
| 29-30 November 2014 | Boka | 14 | 59 | 73 |
| 6-7 December 2014 | Saja | 52 | 19 | 71 |
| 13-14 December 2014 | Kasha | 49 | 19 | 68 |
| 20-21 December 2014 | Tula | 48 | 19 | 67 |
| 28-29 March 2015 | Mera | 51 | 21 | 72 |
